# Enhanced tumor-targeting selectivity by modulating bispecific antibody binding affinity and format valence

**DOI:** 10.1038/srep40098

**Published:** 2017-01-09

**Authors:** Yariv Mazor, Kris F. Sachsenmeier, Chunning Yang, Anna Hansen, Jessica Filderman, Kathy Mulgrew, Herren Wu, William F. Dall’Acqua

**Affiliations:** 1 Department of Antibody Discovery and Protein Engineering, MedImmune, Gaithersburg, MD, USA; 2 Department of Oncology, MedImmune, Gaithersburg, MD, USA; 3 Department of Respiratory, Inflammation and Autoimmunity, MedImmune, Gaithersburg, MD, USA

## Abstract

Bispecific antibodies are considered attractive bio-therapeutic agents owing to their ability to target two distinct disease mediators. Cross-arm avidity targeting of antigen double-positive cancer cells over single-positive normal tissue is believed to enhance the therapeutic efficacy, restrict major escape mechanisms and increase tumor-targeting selectivity, leading to reduced systemic toxicity and improved therapeutic index. However, the interplay of factors regulating target selectivity is not well understood and often overlooked when developing clinically relevant bispecific therapeutics. We show *in vivo* that dual targeting alone is not sufficient to endow selective tumor-targeting, and report the pivotal roles played by the affinity of the individual arms, overall avidity and format valence. Specifically, a series of monovalent and bivalent bispecific IgGs composed of the anti-HER2 trastuzumab moiety paired with affinity-modulated V_H_ and V_L_ regions of the anti-EGFR GA201 mAb were tested for selective targeting and eradication of double-positive human NCI-H358 non-small cell lung cancer target tumors over single-positive, non-target NCI-H358-HER2 CRISPR knock out tumors in nude mice bearing dual-flank tumor xenografts. Affinity-reduced monovalent bispecific variants, but not their bivalent bispecific counterparts, mediated a greater degree of tumor targeting selectivity, while the overall efficacy against the targeted tumor was not substantially affected.

Monoclonal antibodies (mAbs) have become an integral class of biological therapeutics for numerous indications including cancer, autoimmunity, inflammation and metabolic diseases^1^,^2^,^3^,^4^. Yet, despite their remarkable success in the clinic, their monospecific configuration also restricts their overall therapeutic potential as it has become clear that in many disorders, simultaneous deregulation of multiple mediators contribute to the pathology of a disease^5^,^6^,^7^,^8^. Bispecific antibodies (bsAb) by virtue of simultaneously targeting two disease mediators offer greater therapeutic efficacy as well as the capacity to overcome major escape mechanisms evident in mono-targeted therapy^9^,^10^,^11^,^12^. The underlying perception is that dual targeting of antigen double-positive cells over single-positive normal tissues leads to improved target selectivity owing to a strong avidity effect mediated by concurrent binding of the bsAb to both antigens on the surface of the same cell^13^,^14^,^15^,^16^,^17^. It is therefore believed that these new bio-therapeutic agents will open a new era of targeted therapy, providing attractive opportunities of enhanced efficacy coupled with reduced systemic toxicity, leading to an overall improved therapeutic index (TI). However, these arguments are often generalized to all bsAb formats, irrespective of two key design elements associated with the bsAb architecture: (i) the intrinsic binding affinity of the two individual binding arms and (ii) the valence of the two binding domains, namely monovalent *vs*. bivalent. While it has been speculated that affinity-modulation of the separate bsAb arms could minimize normal tissue targeting without impairing the potency against targeted cells^18^,^19^, the interplay of affinity, avidity and format valence in relation to the ability of a bsAb to promote target selectivity remains poorly understood.

We recently reported that dual targeting alone was not sufficient to drive efficient target selectivity^20^. More particularly, we showed that a monovalent bispecific IgG (DuetMab) comprised of an anti-CD70 (2H5)^21^ moiety paired with the high-affinity anti-CD4 ibalizumab^22^ was able to preferentially bind and deplete *in vitro* a target population of CD4^+^/CD70^+^ T cells from a cell mixture containing non-target lymphocytes expressing only one of the target antigens. However, substantial targeting and elimination of non-target CD4^+^/CD70^−^ T cells was still observed. Using an array of affinity-reduced variants of the anti-CD4 mAb, we demonstrated that target selectivity is clearly influenced by the intrinsic affinity of the separate binding arms and can be improved by CDR engineering. Thus, affinity-modulated variants exhibited a greater degree of target selectivity, while the overall efficacy of the bispecific molecule was not compromised^20^.

In this study, we set out to understand how the binding affinity of the individual arms and format valence regulate selective targeting in physiological settings. To that end, we have established a dual-flank tumor xenograft mouse model, carrying human NCI-H358 non-small cell lung cancer (NSCLC) tumors, positive for EGFR and HER2 antigens on one flank, and isogenic NCI-H358 tumors, deficient for HER2 (herein referred to as NCI-H358.HER2.ko) on the opposite flank. The parental cells in this model system represent a double-positive “target tumor” while the single-positive NCI-H358.HER2.ko cells represent a non-target “normal tissue”. Accordingly, we generated a series of bsAbs comprised of the anti-HER2 trastuzumab^23^ moiety paired with an array of affinity-reduced V_H_ and V_L_ regions of the anti-EGFR GA201 mAb^24^. We then assessed the target selectivity of the corresponding anti-EGFR/HER2 bsAb variants, formatted either as monovalent bispecific IgG (DuetMab) or bivalent bispecific in IgG-scFv format^25^ by measuring their ability to selectively target and eradicate the “target tumor” over the non-target “normal tissue” cells on the opposite flank. We provide here for the first time *in vivo* evidence for the pivotal role played by the intrinsic affinity of the separate arms in the ability of a bsAb to confer selective tumor targeting. We further demonstrate the detrimental effect of format valence on the capacity to mediate target selectivity and discuss the implications of our findings in the development of bsAbs optimized for clinical applications.

## Results

### Generation and characterization of NCI-H358.HER2.ko cells

**Figure 1:**
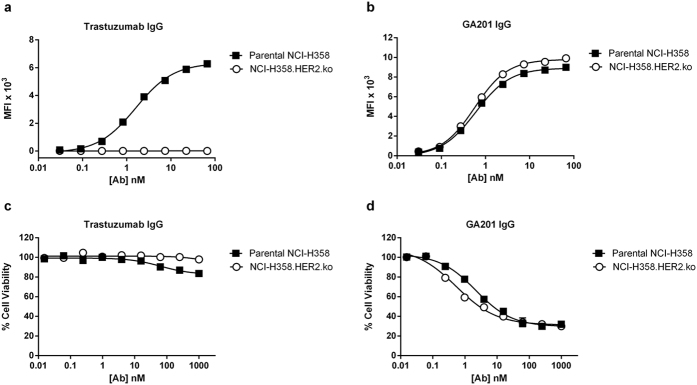
Cell binding and cytotoxic activity of trastuzumab and GA201 as wild type human IgG1 antibodies. (**a**) Cell binding of anti-HER2 trastuzumab. (**b**) Cell binding of anti-EGFR GA201 IgG. (**c**) Cytotoxic activity of trastuzumab. (**d**) Cytotoxic activity of GA201 IgG. Each point represents the mean values of triplicate wells and the ± standard error of the mean (SEM) is represented by error bars.

To investigate how intrinsic affinity of the separate arms and format of a bsAb molecule regulates selective targeting under physiological conditions, we selected the human NCI-H358 cell line as a tumor model and the anti-HER2 trastuzumab and anti-EGFR GA201 as model antibodies. The V_H_ and V_L_ regions of trastuzumab and GA201 were cloned into a mammalian expression vector carrying a wild type human constant heavy gamma 1 (CH1-CH3) and a kappa (κ) constant light (CL) domains and produced as IgG1 antibodies. The intrinsic binding kinetics of the purified anti-HER2 and anti-EGFR IgGs to HER2 and EGFR, respectively, were determined by Octet analysis. As shown in Table 1, the two mAbs exhibited high affinity to their respective antigens. The NCI-H358 cells were chosen as a model system for the following reasons: (i) these cells express similar levels of EGFR and HER2 antigens as determined by receptor density analysis (Table 2) and, hence, should support cross-arm avidity binding analysis and (ii) NCI-H358 cells demonstrate minimal sensitivity to treatment with trastuzumab, yet are quite sensitive to treatment with anti-EGFR mAbs^26^. Since we set out to investigate the impact of affinity-modulation of the EGFR arm on the ability of the bsAb to confer target selectivity, it was essential to select a cell line in which the HER2 receptor will primarily serve as a cell surface anchor for the bsAb to capitalize on cross-arm avidity binding and show no or minimal sensitivity to the HER2 arm alone. For the generation of an isogenic NCI-H358.HER2.ko cell line, we employed the CRISPR/Cas technology^27^. Upon co-transfection of parental cells with a single guide RNA (sgRNA) and Cas9 nuclease, three clones were identified to be deficient of HER2 as determined by genomic sequence analysis (Supplementary Fig. S1). Clone #3-2 was selected for further characterization as it exhibited a comparable growth rate and EGFR receptor density as the parental cell line (Table 2). The cellular binding properties of trastuzumab and GA201 IgGs to parental NCI-H358 and NCI-H358.HER2.ko cells were determined by flow cytometry. As shown in Fig. 1a,b, no detectable cell binding was observed with trastuzumab to the NCI-H358.HER2.ko cells, while the anti-EGFR GA201 IgG demonstrated a similar binding intensity to both cells. The cytotoxic activity of the two mAbs was tested in a cell viability assay to confirm the knock out cells remained insensitive to treatment with trastuzumab and that deletion of the HER2 gene did not in itself affect the sensitivity of the knock out cells to anti-EGFR treatment. As shown in Fig. 1c, the NCI-H358.HER2.ko cells exhibited no sensitivity to treatment with trastuzumab, while mild sensitivity was observed with the parental cells as expected. Interestingly, the sensitivity of the knock out cells to anti-EGFR treatment was marginally enhanced upon deletion of the HER2 gene (Fig. 1d), possibly reflecting the slight increase in EGFR receptor density (Table 2).

### Functional characterization of an anti-EGFR/HER2 DuetMab

**Figure 2:**
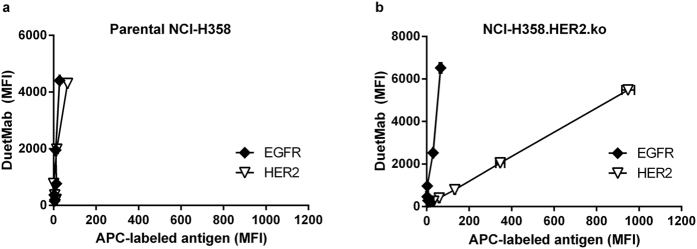
Concurrent binding of DuetMab to EGFR and HER2 receptors on the surface of the same cell. (**a**) Concurrent binding analysis of anti-EGFR/HER2 DuetMab to parental NCI-H358 cells. (**b**) Concurrent binding analysis of anti-EGFR/HER2 DuetMab to NCI-H358.HER2.ko cells. To determine concurrent binding of EGFR and HER2 receptors by cell-bound DuetMab, each cell population was examined individually using fluorescent dye labeled EGFR and HER2 antigens. (Y-axis), Detection of cell-bound DuetMab. (X-axis), Detection of free antigen binding arms on cell-bound DuetMab. Data points refer to serial dilutions of the DuetMab starting at a sub-saturating concentration of 1.2 nM. Each point represents the mean values of duplicate wells and the ± standard error of the mean (SEM) is represented by error bars.

**Figure 3:**
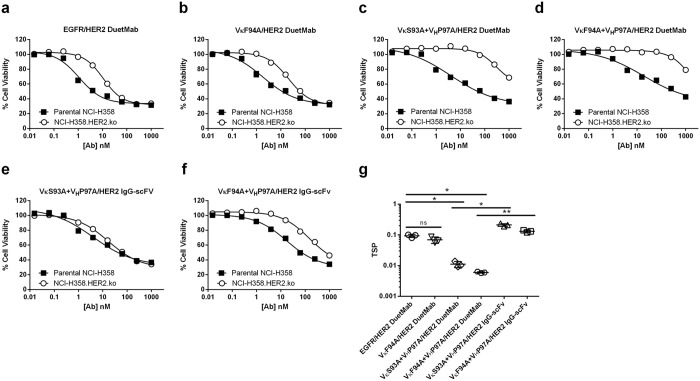
Selective cell cytotoxicity of EGFR affinity-modulated bsAb variants in a cell mixture containing target and non-target cells. (**a**) Selective cytotoxic activity of parental EGFR/HER2 DuetMab. (**b**) Selective cytotoxic activity of VκF94A/HER2 DuetMab variant. (**c**) Selective cytotoxic activity of VκS93A + V_H_P97A/HER2 DuetMab variant. (**d**) Selective cytotoxic activity of VκF94A + V_H_P97A/HER2 DuetMab variant. (**e**) Selective cytotoxic activity of VκS93A + V_H_P97A/HER2 IgG-scFv variant. (**f**) Selective cytotoxic activity of VκF94A + V_H_P97A/HER2 IgG-scFv variant. (**g**) Mean of TSP values based upon data from three independent experiments. To determine selective cell targeting and elimination, the double-positive NCI-H358 cells and single-positive NCI-H358.HER2.ko cells were pre-stained with different tracer dyes, combined at equal ratios and incubated with serial dilutions of the various bsAbs. Selective cytotoxic activity was determined by cell imaging cytometry. Each point on the graphs represents the mean values of triplicate wells and the ± standard error of the mean (SEM) is represented by error bars. Statistical significance was determined by One way ANOVA for multiple comparisons (P < 0.05 *P < 0.01 **P < 0.001 ***P < 0.0001 ****ns: not significant).

To evaluate the functional properties of an anti-EGFR/HER2 bsAb, the variable domains of trastuzumab and GA201 mAbs were formatted into our previously described monovalent bispecific DuetMab platform (Supplementary Fig. S2)^26^. The corresponding anti-EGFR/HER2 DuetMab carrying a wild type human γ1 heavy chain was produced from mammalian cells as previously described^26^. The purity and oligomeric state of the DuetMab were assessed by a BioAnalyzer and size exclusion chromatography (SEC) (Supplementary Fig. S3). The expected mass and homogeneity of the intact molecule were confirmed by liquid chromatography-mass spectrometry (LC-MS) (Supplementary Fig. S3). We then tested whether the DuetMab can concurrently bind EGFR and HER2 antigens on the surface of the same cell and thereby mediate cross-arm avidity binding. This experiment is imperative when investigating the ability of a bsAb to promote selective targeting since functional avidity targeting is mediated by concurrent engagement to two antigens on the surface of the same cell. To detect free antigen-binding arms on cell-bound DuetMab, we used fluorescent dye labeled soluble EGFR and HER2 proteins. As shown in Fig. 2a, when the DuetMab was incubated with the double-positive NCI-H358 cells, no detectable fluorescent signal was observed following the addition of labeled EGFR or HER2 proteins, indicating that both arms of the DuetMab are concurrently bound to the cell. In contrast, incubation of the DuetMab with the NCI-H358.HER2.ko cells resulted in a concentration dependent increase in fluorescent signal following the addition of the labeled HER2 protein but not with the labeled EGFR (Fig. 2b), confirming the EGFR arm of cell-bound DuetMab was bound monovalently. To assess whether cross-arm binding promotes target selectivity, we tested the ability of the DuetMab to selectively target and eliminate the double-positive NCI-H358 cells from a cell mixture also containing the NCI-H358.HER2.ko cells. The two cell populations were pre-stained with different tracer dyes, combined at a 1:1 ratio and then interacted with serial dilutions of the DuetMab. Selective cytotoxic activity was analyzed by cell imaging cytometry on a Celigo instrument. To facilitate comparative analysis of the data, we introduced a “target selectivity parameter” (TSP), determined by dividing the half maximal inhibitory concentration (IC_50_) value obtained against the parental cells with the IC_50_ value obtained for the HER2 knock out cells (Table 3). In this case, a TSP value lower than 1.0 is indicative of selective targeting. As shown in Fig. 3a, the DuetMab exhibited a greater degree of cytotoxicity against the double-positive over single-positive cells as indicated by IC_50_ values of 1.08 nM *vs.* 10.71 nM, respectively, generating a TSP value of 0.1. Nevertheless, substantial targeting and elimination of HER2 knock out cells still occurred as a result of monovalent DuetMab interactions. Particularly at saturating antibody concentrations (≥100 nM), both cell populations exhibited similar residual cell-viability. These results are in alignment with our previous findings for the CD4/CD70 DuetMab^20^ and correlate well with observations recently reported for other dual-targeting bsAb agents^15^,^18^,^28^. While the investigators in these studies have demonstrated enhanced targeting of double-positive over single-positive cells, substantial targeting of non-target, single-positive cells was also reported. Taken together, our findings demonstrate that significant eradication of non-target cells can result from monovalent, single-arm binding provided the affinity of the single arm is high enough.

### Generation and characterization of affinity-modulated anti-EGFR/HER2 DuetMab variants

To determine how the intrinsic binding affinity of the anti-EGFR arm regulates target selectivity, we constructed an array of affinity-reduced variants of the GA201 mAb by employing alanine mutagenesis to exposed residues in complementarity-determining region (CDR)H3 and L3^29^. The variable domains of three variants exhibiting a ~10–250-fold reduction in affinity compared with the parental sequence were paired with the trastuzumab arm and converted into a DuetMab format. The corresponding DuetMab variants were produced from mammalian cells and their oligomeric state and purity were determined as described above for the parental DuetMab (Supplementary Fig. S3). As shown in Table 1, the affinity-reduced DuetMab variants retained the relative intrinsic affinity and ranking of the IgGs from which they were derived as determined by Octet analysis. Notably, no significant difference was identified between the association-rates (*K*_*on*_) of the affinity-modulated variants and the parental GA201 sequence, while up to two-log reduction in the dissociation-rates (*K*_*off*_) were measured. To assess whether affinity-modulation of the anti-EGFR arm leads to improved target selectivity, we tested the ability of the DuetMab variants to induce selective targeting and elimination of the parental NCI-H358 cells using the same cell mix as described above. As shown in Fig. 3b–d and G, the EGFR affinity-reduced DuetMab variants mediated a greater degree of selectivity compared with the parental DuetMab as reflected by preferential killing of the double-positive over the single-positive cells. As shown in Table 3, while the overall potency of the affinity-reduced variants against the parental NCI-H358 cells was moderately impaired, as indicated by an increase in the IC_50_ values, variants VκS93A + V_H_P97A and VκF94A + V_H_P97A that displayed ~50- and 250-fold reduced affinity, respectively, exhibited significantly improved target selectivity as reflected by mean TSP values of 0.01 (*P* < 0.015) and 0.006 (*P* < 0.019), respectively. This improved selectivity is attributed to the strong avidity mediated by concurrent binding of the DuetMab to EGFR and HER2 on the surface of the same cell. We confirmed the ability of the affinity-modulated DuetMab variants to cross-bind EGFR and HER2 antigens on the cell surface in an avid manner by comparing the half-maximal effective concentration (EC_50_) values generated against the parental NCI-H358 cells with EC_50_ values generated against the NCI-H358.HER2.ko cells. As shown in Table 1 and (Supplementary Fig. S4), the affinity-reduced variants displayed lower EC_50_ values upon binding to the double-positive NCI-H358 cells compared with EC_50_ values obtained for the single-positive HER2 knock out cells. Our results are in agreement with recent reports, demonstrating that, depending on the spatial arrangement of the antigen binding sites as well as the antigen surface distribution and density, significant functional avidity may be triggered by cross-arm binding^14^,^16^,^20^,^30^. Remarkably, a 10-fold reduction in affinity of the EGFR arm was not sufficient to significantly enhance target selectivity over that obtained with the parental DuetMab, as evidenced by a mean TSP value of 0.07 acquired for variant VκF94A. Taken together, our data show that the target selectivity of a monovalent bispecific IgG is directly influenced by the intrinsic affinity of the individual binding arms.

### EGFR affinity-reduced DuetMabs exhibit improved tumor-targeting selectivity *in vivo*

**Figure 4:**
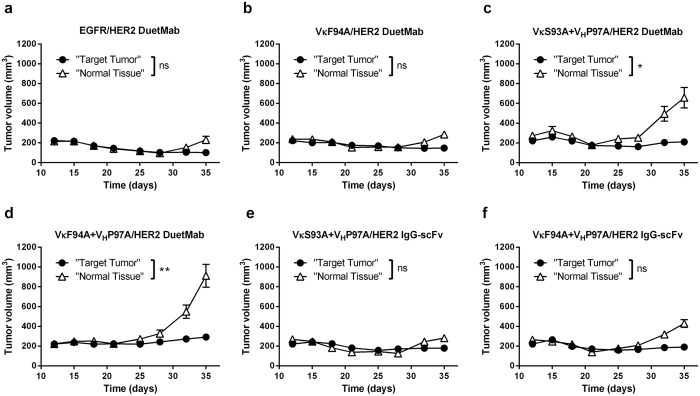
Tumor-targeting selectivity of EGFR affinity-modulated bsAb variants in nude mice bearing dual-flank tumor xenografts. (**a**) Selective tumor-targeting activity of parental EGFR/HER2 DuetMab. (**b**) Selective tumor-targeting activity of VκF94A/HER2 DuetMab variant. (**c**) Selective tumor-targeting activity of VκS93A + V_H_P97A/HER2 DuetMab variant. (**d**) Selective tumor-targeting activity of VκF94A + V_H_P97A/HER2 DuetMab variant. (**e**) Selective tumor-targeting activity of VκS93A + V_H_P97A/HER2 IgG-scFv variant. (**f**) Selective tumor-targeting activity of VκF94A + V_H_P97A/HER2 IgG-scFv variant. To determine tumor-targeting selectivity, the double-positive NCI-H358 cells as “target tumor” and single-positive NCI-H358.HER2.ko cells as “normal tissue” were grafted on opposing flanks of athymic nude mice. Dual-flank tumor bearing mice were treated twice weekly with the various bsAbs for a total of 8 treatments and selective tumor growth inhibition was measured twice weekly. Tumor size represents the mean values of 8 mice per treatment group and the ± standard error of the mean (SEM) is represented by error bars. Statistical comparisons were conducted by calculating the area under the curve (AUC) of paired “target” and “non-target” tumors, and significance of tumor selectivity was determined by paired t-test (P < 0.05 *P < 0.01 **P < 0.001 ***P < 0.0001 ****ns: not significant).

To determine whether the improved target selectivity seen *in vitro* could be exploited to enhance functional selectivity in physiological settings, we tested the ability of the affinity-modulated DuetMab variants to selectively target and eradicate double-positive NCI-H358 tumors over single-positive NCI-H358.HER2.ko tumors in nude mice bearing dual-flank tumor xenografts. In this model system, the parental cells represented a “target tumor” expressing both target antigens, while the isogenic HER2 knock out cells represented non-target, single-positive cells or, as referred to herein by analogy, “normal tissue”. In this instance, the non-target tumor may exemplify the skin tissue. EGFR plays a key role in skin biology, and signaling through this receptor is shown to be crucial for the normal development and physiology of the epidermis^31^,^32^. Thus, treatment of cancer patients with anti-EGFR bio-therapeutics is commonly associated with a set of unique dermatological toxicities which affect patients’ quality of life and often lead to dose reduction or discontinuation of treatment^33^,^34^,^35^,^36^. To establish dual-flank tumor xenografts, parental NCI-H358 and NCI-H358.HER2.ko cells were grafted on the opposing flanks of BALB/c athymic nude mice. Dual-flank tumor bearing mice were then treated with DuetMab variants at 5 mg/kg, twice weekly for a total of 8 doses and tumor growth inhibition (TGI) was monitored. As shown in Fig. 4, under physiological conditions, the parental DuetMab exhibited insignificant tumor targeting selectivity, with comparable TGI induced against the double-positive “target tumor” and single-positive “normal tissue”. In contrast, EGFR affinity-reduced DuetMabs and in particular variants VκS93A + V_H_P97A and VκF94A + V_H_P97A, mediated a significant degree of selective tumor targeting (*P* < 0.014 and *P* < 0.003, respectively), as evidenced by preferential eradication of the double-positive over the single-positive tumors. Consistent with our *in vitro* data, the level of tumor selectivity was inversely proportional to the reduced intrinsic affinity to EGFR. Notably, variant VκF94A + V_H_P97A, which had the lowest intrinsic EGFR affinity of ~150 nM (~250-fold less than parental DuetMab), induced the most significant tumor selectivity, while no substantial reduction in potency against the “target tumor” was observed. Again, this enhanced tumor selectivity is indicative of a strong avidity mediated by cross-binding of EGFR and HER2 antigens on double-positive tumors. However, since no avidity binding is occurring on HER2 knock out tumors, the substantial reduction in EGFR affinity has a negative effect on monovalent interaction and targeting of single-positive tumors. By contrast, variant VκF94A, displaying a 10-fold reduction in EGFR affinity, was inefficient in discriminating between the “target tumor” and “normal tissue” and overall exhibited similar target selectivity properties as the parental DuetMab.

**Figure 5:**
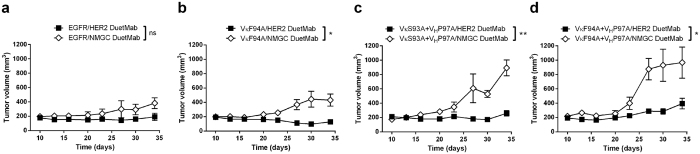
Antitumor activity of monospecific and bispecific EGFR affinity-modulated variants in nude mice bearing single-flank NCI-H358 xenografts. (**a**) Antitumor activity of parental GA201 formatted as either bispecific anti-EGFR/HER2 DuetMab or monospecific anti-EGFR/NMGC DuetMab. (**b**) Antitumor activity of variant VκF94A formatted as either bispecific anti-EGFR/HER2 DuetMab or monospecific anti-EGFR/NMGC DuetMab. (**c**) Antitumor activity of variant VκS93A + V_H_P97A formatted as either bispecific anti-EGFR/HER2 DuetMab or monospecific anti-EGFR/NMGC DuetMab. (**d**) Antitumor activity of variant VκF94A + V_H_P97A formatted as either bispecific anti-EGFR/HER2 DuetMab or monospecific anti-EGFR/NMGC DuetMab. Single-flank tumor bearing mice were treated twice weekly with monospecific and bispecific formats of the EGFR affinity-modulated variants for a total of 8 treatments and tumor growth inhibition was measured twice weekly. Tumor size represents the mean values of 5 mice per treatment group and the ± standard error of the mean (SEM) is represented by error bars. Statistical comparisons were conducted by calculating the area under the curve (AUC) comparing each monospecific DuetMab with its corresponding bispecific DuetMab. Statistical significance was determined by unpaired t-test (P < 0.05 *P < 0.01 **P < 0.001 ***P < 0.0001 ****ns: not significant).

To confirm that the improved tumor-targeting selectivity observed with the affinity-modulated DuetMab variants was directly influenced by the affinity of the EGFR arm and not due to differences in the intrinsic sensitivity of parental NCI-H358 and NCI-H358.HER2.ko tumors to anti-EGFR treatment, we generated monospecific formats of the DuetMab variants by pairing the Fab domains of the EGFR affinity-reduced variants with a Fab of an isotype control (‘NMGC’). We then compared the TGI induced by these affinity-modulated monospecific EGFR/NMGC DuetMabs with the TGI mediated by their corresponding bispecific EGFR/HER2 DuetMab counterparts in single-flank tumor xenografts carrying only the double-positive NCI-H358 tumors. As shown in Fig. 5, the high-affinity parental GA201 as monospecific EGFR/NMGC DuetMab induced a potent TGI, insignificantly different from the TGI induced by the corresponding bispecific EGFR/HER2 DuetMab. These data confirm that extensive eradication of double-positive tumors can be induced by monovalent binding provided the affinity of the single arm is high enough. In contrast, the affinity-reduced variants as monospecific EGFR/NMGC DuetMabs were significantly inferior in their TGI activity (*P* < 0.013 and *P* < 0.002, respectively), compared to their bispecific DuetMab counterparts. Consistent with the dual-flank tumor xenograft system, the TGI activity mediated by the monospecific DuetMab variants was inversely proportional to the reduced affinity to EGFR. Collectively, our data show that under physiological conditions, the tumor-targeting selectivity of monovalent bsAbs is clearly influenced by the intrinsic affinity of the separate binding arms.

### Effect of avidity and format valence on tumor-targeting selectivity

To comprehend the role of format valence in the ability of a bsAb to endow tumor-targeting selectivity, we formatted variants VκS93A + V_H_P97A and VκF94A + V_H_P97A that as monovalent bispecific DuetMabs, mediated a significant degree of selective tumor-targeting, into a bivalent bispecific IgG-scFv format as first described by Coloma and Morrison (Supplementary Fig. S2)^25^. Here, the variable domains of trastuzumab were fused, as single-chain fragment variable (scFv), to the C-terminus of GA201 mAb heavy chains, generating a tetravalent molecule comprised of two natural Fab domains targeting EGFR plus two scFv-binding sites for HER2 antigen. We then compared the targeting selectivity of the monovalent and bivalent bispecific formats in targeting and eliminating the double-positive NCI-H358 cells over the single-positive NCI-H358.HER2.ko; first, in a cell mixture and then in the dual-flank tumor xenograft system. As shown in Fig. 3e,f, when in bivalent bispecific IgG-scFv format, variants VκS93A + V_H_P97A and VκF94A + V_H_P97A induced similar cytotoxic activity against the parental NCI-H358 cells as their corresponding monovalent bispecific DuetMab counterparts. As shown in Table 3, the two bispecific formats obtained comparable IC_50_ values, indicating that an increase in valence has no effect on the cytotoxic potency mediated against the double-positive target cells. However, contrary to the significant target selectivity seen with the DuetMab formats, the two variants as IgG-scFv, exhibited poor cytotoxic selectivity as reflected by substantial targeting and elimination of the non-target NCI-H358.HER2.ko cells, yielding mean TSP values of 0.21 and 0.13, respectively. This poor target selectivity can be attributed to the strong avidity mediated by bivalent binding of the IgG-scFv variants to EGFR on the surface of NCI-H358.HER2.ko cells. As shown in Table 1 and (Supplementary Fig. S4) the two bivalent bispecific variants displayed similar EC_50_ values against the single-positive NCI-H358.HER2.ko cells and double-positive parental NCI-H358 cells. Similarly, as shown in Fig. 4e,f, when tested *in vivo* in the dual-flank tumor xenograft system, the bivalent bispecific variants and, in particular, variant VκS93A + V_H_P97A, exhibited insignificant tumor-targeting selectivity as reflected by comparable TGI mediated against the “target tumor” and “normal tissue”. Evidently, the reformation of the two variants into a bivalent bispecific format resulted in a loss of their previously described improved tumor-targeting selectivity. Taken together, these results suggest that an increase in binding sites valence (that is, avidity) may correspond with enhanced reactivity against non-target cells, which may lead to elevated systemic toxicity and overall reduced therapeutic index. Therefore, bivalent bispecific formats are likely to be less efficient in promoting improved target selectivity compared to monovalent bispecific formats.

## Discussion

Bispecific antibodies are emerging as a leading class of biological therapeutics. Their capacity to simultaneously target multiple disease pathways opens attractive new perspectives on the basis of efficacy and selectivity that potentially lead to better drug safety and improved therapeutic index. The excitement in this area is attested by a surge of more than 60 different bispecific antibody formats reported to date and the progression of over 30 new bsAb compounds into clinical development^12^. However, these formats not only vary in their molecular weight, pharmacokinetics and the ability to support immune effector functions, but most importantly, significantly differ in their geometry, number of antigen-binding sites and the intrinsic affinity of the individual arms. In this respect, we argue that the interplay of affinity, avidity and format valence in relation to the capacity of a bsAb to confer target selectivity is oft-overlooked when developing clinically relevant bispecific therapeutics. In this study, we systemically interrogated the collective role of affinity, avidity and format valence in the ability of a selection of monovalent and bivalent bsAbs composed of the anti-HER2 trastuzumab moiety paired with an array of affinity-modulated variants of the anti-EGFR GA201 mAb to promote selective tumor-targeting under physiological conditions. Using a dual-flank tumor xenograft model system, carrying a double-positive “target tumor” on one flank, and an isogenic HER2 knock out, single-positive tumor as “normal tissue” on the opposite flank, we demonstrated that: 1) dual-targeting alone is not sufficient for effective tumor-targeting selectivity; 2) tumor-targeting selectivity is clearly influenced by the intrinsic affinity of the individual binding arms; 3) affinity-modulation of monovalent bispecific IgG separate arms could significantly limit normal tissue targeting without impairing the potency against the targeted tumor; and 4) an increase in binding sites valence has a detrimental effect in the ability of a bsAb to endow selective tumor-targeting. The objective of this study was not to develop a new bispecific therapeutic agent with improved *in vivo* anti-tumor activity, but rather, to dissect the interplay of factors that regulate dual-targeting selectivity. Nevertheless, simultaneous targeting of EGFR and HER2 is postulated to have therapeutic benefits, since overexpression, mutations or inappropriate signaling through these receptors were shown to play critical roles in tumor progression and metastasis, and confer poor prognosis in various cancers^37^,^38^,^39^,^40^,^41^. In addition, the two pathways are known to be bypassed in monotherapy and resistance mechanisms often emerge upon single targeting^42^.

To our knowledge, this is the first *in vivo* study that has demonstrated the interplay of affinity and format valence on the capacity of a bsAb to selectively target and eradicate double-positive target tumors over single-positive, isogenic non-target tumors. Our dual-flank tumor xenograft model provides a predictive examination of selective targeting and drug toxicity as it simulates a physiological environment in which target and non-target cells co-exist. This is especially relevant in cancer, as most target antigens currently pursued by antibody therapy are not necessarily “tumor specific” but rather “tumor-associated”; that is, they are also expressed in normal, non-malignant tissue^43^,^44^. In this regard, the single-positive NCI-H358.HER2.ko tumors used in this study are analogous to skin that is sensitive to anti-EGFR treatment^31^,^32^,^33^,^34^,^35^,^36^. It has been speculated that since malignant cells frequently express higher densities of the target antigen compared to normal cells, as long as the intrinsic affinity of the individual arms is not remarkably high, dual-target avidity binding will drive preferential targeting and accumulation of the bsAb onto double-positive target cells over single-positive normal tissue^19^. However, in some malignancies the target antigen is expressed at densities not significantly different than those found on normal healthy cells^45^. In addition, a growing body of evidence suggests high intratumor heterogeneity at the expression levels of many target antigens^46^,^47^,^48^. The low densities of EGFR and HER2 displayed on the NCI-H358 cancer cells used in this study strongly suggest that efficient tumor selectivity by dual-target avidity binding is not contingent upon overexpression of the two target antigens relative to healthy tissue, but rather primarily dependent on the intrinsic affinity of the two separate arms and the valence of the binding domains. These findings provide attractive new opportunities for the use of affinity-modulated monovalent bsAbs in cancer therapy, as they facilitate the recruitment of a broader set of target antigens, previously considered unattractive for therapeutic applications due to comparable densities to those found on normal healthy cells. Naturally, as with any dual targeting strategy, it remains subject to the caveat that a tumor can potentially escape therapy due to downregulation or mutation of either target antigen, creating antigen-loss escape variants^49^,^50^.

In conclusion, the results of our study provide a deeper understanding of the factors that regulate dual-targeting selectivity and underline the pivotal roles played by the affinity of the individual arms, the overall avidity and format valence in the capacity of a bsAb to promote selective tumor targeting. We propose a careful examination of these key design parameters when developing clinically optimized bispecific therapeutics. Safety studies in non-human primates using affinity-optimized naked DuetMabs, as well as antibody-drug conjugate DuetMabs, are in progress to determine improvements in the therapeutic index.

## Methods

### Generation of HER2 KO NCI-H358 cells

The human tumor cell line, NCI-H358 was obtained from the American Type Culture Collection (ATCC). Cells were routinely cultured in RPMI-1640 with GlutaMAX supplemented with 10% HI FBS at 37 °C and 5% CO_2_ in a humidified incubator. NCI-H358.HER2.ko cells were generated by PNA Bio using the CRISPR/Cas technology essentially as described^27^. Briefly, parental NCI-H358 cells were co-transfected with 2 μg each of plasmid expressing sgRNA CTCCATTGTCTAGCACGGCC targeting exon 5 of HER2 and Cas9 expression vector using Lipofectamine 2000 (Life Technologies). After 48 hours, cells were plated by limited dilution for single cell cloning. Total DNA was extracted from individual clones and PCR was performed using primers 5′ AAAAATTTGCAGACGCCATC (forward) and 5′ CCTGCATTCTCACGATTGAA (reverse). The PCR products were then digested with T7E1 enzyme to detect for insertions/deletions events. Following genomic sequence analysis 3 clones were identified as deficient of HER2. The population doubling level (PDL) of parental NCI-H358 cells and NCI-H358.HER2.ko clones was determined using the following formula: PDL = 3.32 × (log n2 – log n1) + X, where n1 = the cell number at the beginning of the incubation, n2 = the cell number at the end of incubation time, and X = the starting doubling level of the population being quantitated.

### Antibody mutagenesis and production

Alanine mutagenesis of targeted residues in CDRH3 and L3 of the anti-EGFR GA201 mAb was performed by site-directed mutagenesis using standard PCR techniques essentially as described^29^. For production as human IgG1, V_H_ and V_L_ DNA segments were cloned into an Orip/EBNA-1-based episomal mammalian expression plasmid, pOE as described^51^. DuetMab and Bs3Ab bispecific antibodies were constructed and produced essentially as described^26^,^51^, respectively. All antibodies were transiently expressed in HEK293F cells using 293fectin™ (Invitrogen) and grown in serum-free Freestyle™ medium (Invitrogen) according to the supplier’s recommended procedures. Cell culture supernatants were harvested 10 days after transfection and filtered through a 0.22 μm sterile filter. Antibody concentration in culture supernatants was measured by an Octet384 instrument (ForteBio) according to the manufacturer’s protocol. Antibodies were purified by protein A affinity chromatography on a MabSelect SuRe column (GE Healthcare) and subsequently buffer-exchanged in phosphate buffered saline (PBS) pH 7.2. Protein aggregates were removed by size exclusion chromatography (SEC) using a Superdex 200 column (GE Healthcare). Monomeric antibody fractions were pooled and stored as 1.0 mg/mL aliquots at −80 °C. The concentration of purified proteins was determined by their absorbance at 280 nm. The purity and oligomeric state of purified molecules was determined by SEC and BioAnalizer (Agilent) and the expected mass was confirmed by liquid chromatography-mass spectrometry (LC-MS).

### Binding kinetics measurements

Kinetic measurements to soluble forms of EGFR (R&D Systems) and HER2 (eBioscience) ligands were measured by biolayer interferometry on an Octet384 instrument (ForteBio) essentially as described^20^. Briefly, for assessment of intrinsic binding affinity of IgGs and IgG-scFvs, purified antibodies at 10 μg/mL in PBS pH 7.2, 3 mg/mL BSA, 0.05% (v/v) tween 20 (assay buffer) were captured on anti-human IgG Fc biosensors (ForteBio). The loaded biosensors were washed with assay buffer to remove any unbound protein before carrying out association and dissociation measurements with serial dilutions of the antigen ligands. For determination of intrinsic kinetics of DuetMab antibodies, streptavidin biosensors (ForteBio) were loaded with biotinylated EGFR at 5 μg/mL in assay buffer. Following a washing step, association and dissociation measurements were carried out using serial dilutions of the purified DuetMabs. Kinetic parameters (k_on_ and k_off_) and dissociation constant (K_D_) were calculated from a non-linear fit of the data using the Octet384 software v.7.2.

### Receptor density analysis

Receptor density studies were performed by flow cytometry on a MACSQuant VYB (Miltenyl Biotec) essentially as described^20^. Briefly, anti-EGFR GA201 and anti-HER2 trastuzumab IgGs were first labeled with Alexa Fluor 647 labeling kit (Invitrogen) according to the supplier’s recommendations. Antibody concentration and fluorochrome to protein (F:P) ratio were determined by a ND-1000 spectrophotometer (NanoDrop). Parental NCI-H358 and NCI-H358.HER2.ko cells at ~4 × 10^6^ cells/mL were washed with ice-cold FACS Buffer (PBS pH 7.2, 2% FBS, 2 mM EDTA and 0.1% sodium azide) followed by incubation with the conjugated antibodies at saturating concentrations (≥ 20 μg/mL) for 30 min at 4 °C. After washing with FACS buffer, cells were fixed in ice-cold 1.8% paraformaldehyde (PFA) and detection of bound antibodies was performed on MACSQuant VYB using MACSQuantify™ software. Results were analyzed using the FlowJo analysis software (Tree Star). For quantitation of EGFR and HER2 receptor density on cells, Quantum Alexa Fluor 647 MESF (Molecules of Equivalent Soluble Fluorochrome) beads (Bangs Laboratories) were analyzed on the flow cytometer using similar settings to establish a standard curve. Using the QuickCal program (Bangs Laboratories) the calculated MESF was then divided by the antibody F:P ratio to give a corrected Antibody Binding Capacity (ABC).

### Cell binding assays

Cellular binding analyses were performed by flow cytometry using a LSR II (Becton Dickinson) instrument essentially as described^20^. For direct cell binding, ~5 × 10^4^ cells/well were washed twice with PBS pH 7.2, 2% FBS, 2 mM EDTA and 0.1% sodium azide (FACS buffer) followed by incubation with serial dilutions of the tested IgGs for 1 h at 4 °C. After washing with FACS buffer, FITC-conjugated goat anti-human Fcγ (Jackson ImmunoResearch) was added for 45 min at 4 °C for detection of cell-bound IgG. Half-maximal effective concentration (EC_50_) values were used to estimate apparent cell binding affinities. To determine concurrent binding of EGFR and HER2 receptors by cell-bound DuetMab, parental NCI-H358 and NCI-H358.HER2.ko cells were incubated with serial dilutions of the anti-EGFR/HER2 DuetMab starting at a sub-saturating concentration of 1.2 nM for 1 hour at 4 °C. After washing with FACS buffer, cell-bound DuetMab was detected by PE-conjugated goat anti-human Fcγ (Jackson ImmunoResearch) and free antigen binding arms on cell-bound DuetMab were detected by biotinylated soluble EGFR and HER2 proteins at 5 nM followed by Streptavidin-Allophycocyanin (APC) (Biolegend). Data analysis was performed using the FlowJo software and the mean fluorescence intensity (MFI) was used to determine binding intensity. Based on physical properties (height, width and density) only single cells were gated for analysis.

### Cell viability assays

Cell cytotoxicity studies were performed on a Celigo S Cell Imaging Cytometer (Nexcelom BioScience). To determine selective cytotoxicity, parental NCI-H358 and HER2 KO cells were first stained with CellTracker™ Green CMFDA and CellTracker™ Violet BMQC (Thermo Fisher Scientific), respectively, according to the manufacturer’s instructions. Cells were then combined at 1:1 ratio in RPMI-1640 with GlutaMAX supplemented with 10% HI FBS and seeded in 96-well plates at a density of 1 × 10^4^ cells/well. Antibodies at various concentrations were added to triplicate samples, and the cells were incubated for 72 hours at 37 °C and 5% CO_2_ in a humidified incubator. To enumerate each cell population, bright field, green fluorescence and blue fluorescence channels were used. Overlay of bright field with either green fluorescence or blue fluorescence identified and quantified CMFDA or BMQC positive cells using Celigo image processing software. % cell viability was calculated based on the change in cell number relative to a no antibody treatment control.

### *In vivo* tumor xenograft models

To establish dual-flank tumor xenografts, BALB/c athymic nude mice, 5–6 weeks old (Envigo) were injected subcutaneously with 5 × 10^6^ parental NCI-H358 cells into the left flank and 5 × 10^6^ NCI-H358.HER2.ko cells into the right flank. When tumors reached approximately 160 mm^3^, mice were randomized and sorted into study groups of 8 animals. Antibodies at 5 mg/kg were dosed intraperitoneally twice weekly for a total of 8 treatments. Tumors were measured twice weekly using calipers and tumor volume was calculated as [L(length) × W^2^(width)]/2. Single-flank tumor xenografts were established by subcutaneous implantation of 5 × 10^6^ parental NCI-H358 cells into the left flank of mice, 5 animals per study group and followed the same procedure as described above. Animals were housed in a United States Department of Agriculture (USDA) registered and Association for Assessment and Accreditation of Laboratory Animal Care (AAALAC) accredited animal facility in accordance with the guide for care and use of laboratory animals. All experiments were performed according to the guidelines approved by the MedImmune Institutional Animal Care and Use Committee (IACUC).

### Statistical analysis

Graphical and statistical analyses were carried out using Graph Pad Prism software. Significant differences were determined by One way ANOVA followed by Holm-Sidak’s multiple comparisons test, unpaired and paired t-test as specified. Statistical significance was accepted for *P* values < 0.05 at 95% confidence interval.

## Additional Information

**How to cite this article**: Mazor, Y. *et al*. Enhanced tumor-targeting selectivity by modulating bispecific antibody binding affinity and format valence. *Sci. Rep.* **7**, 40098; doi: 10.1038/srep40098 (2017).

**Publisher's note:** Springer Nature remains neutral with regard to jurisdictional claims in published maps and institutional affiliations.

## Supplementary Material

Supplementary Information

## Figures and Tables

**Table 1:** Intrinsic and apparent binding affinity to target antigens.

Antibody	Intrinsic binding kinetics determined by Octet^c^			Apparent cell binding affinity parental NCI-H358^d^	Apparent cell binding affinity NCI-H358.HER2.ko^d^
	K_on_ (M^−1^ s^−1^)	K_off_ (s^−1^)	K_D_ (nM)	EC_50_ (nM)	EC_50_ (nM)
Trastuzumab IgG^a^	2.3 × 10^5^	2.6 × 10^−4^	1.1	1.6	ND
GA201 IgG^b^	2.5 × 10^5^	1.6 × 10^−4^	0.6	0.5	0.5
VκF94A IgG^b^	2.0 × 10^5^	1.4 × 10^−3^	7.2	ND^e^	ND
VκS93A + V_H_P97A IgG^b^	1.4 × 10^5^	3.3 × 10^−3^	24	ND	ND
VκF94A + V_H_P97A IgG^b^	1.1 × 10^5^	1.6 × 10^−2^	148	ND	ND
EGFR/HER2 DuetMab^b^	2.3 × 10^5^	1.7 × 10^−4^	0.7	0.9	1.1
VκF94A/HER2 DuetMab^b^	2.2 × 10^5^	1.8 × 10^−3^	8.1	1.7	2.3
VκS93A + V_H_P97A/HER2 DuetMab^b^	1.8 × 10^5^	5.7 × 10^−3^	32	5.6	>50
VκF94A + V_H_P97A/HER2 DuetMab^b^	1.4 × 10^5^	2.3 × 10^−2^	167	8.3	>200
VκS93A + V_H_P97A/HER2 IgG-scFv^b^	1.5 × 10^5^	4.1 × 10^−3^	27	5.1	6.5
VκF94A + V_H_P97A/HER2 IgG-scFv^b^	1.2 × 10^5^	1.9 × 10^−2^	159	7.7	9.8

The amino acid residues in variable regions (V_H_ or V_L_) are numbered by Kabat numbering system.

^a^Binding measured against HER2.

^b^Binding measured against EGFR.

^c^Kinetic measurements to soluble monomeric forms of EGFR and HER2 were performed using an Octet384 instrument. The dissociation constants, K_D_, were calculated as the ratio of k_off_/k_on_ from a non-linear fit of the data.

^d^Apparent cell binding affinities to parental NCI-H358 and NCI-H358.HER2.ko cells were determined by flow cytometry. Half maximal effective concentration (EC_50_) values were calculated as the antibody concentration that generates 50% of the maximal MFI signal.

^e^Not determined.

**Table 2:** Doubling rate and receptor density properties of parental NCI-H358 and NCI-H358.HER2.ko tumor cell-lines.

Cell	PDL^a^	EGFR	HER2
Parental NCI-H358	40.0	3.1 × 10^4^	2.8 × 10^4^
NCI-H358.HER2.ko clone #3-2	40.6	3.7 × 10^4^	U^b^
NCI-H358.HER2.ko clone #3-4	39.3	5.3 × 10^4^	U^b^
NCI-H358.HER2.ko clone #54	41.1	2.9 × 10^4^	U^b^

^a^Population doubling level, (hours).

^b^U: Undetectable.

**Table 3:** Cell cytotoxicity and calculated target selectivity parameter (TSP) values of anti-EGFR/HER2 bsAb variants.

Cell	Parental NCI-H358	NCI-H358.HER2.ko	
Antibody	IC_50_ (nM)	IC_50_ (nM)	Mean TSP^a^
EGFR/HER2 DuetMab	1.1	10.7	0.09 ± 0.006
VκF94A/HER2 DuetMab	1.8	20.9	0.07 ± 0.009
VκS93A + V_H_P97A/HER2 DuetMab	4.1	296	0.01 ± 0.001
VκF94A + V_H_P97A/HER2 DuetMab	18.6	3251	0.006 ± 0.0002
VκS93A + V_H_P97A/HER2 IgG-scFv	4.0	17.0	0.21 ± 0.012
VκF94A + V_H_P97A/HER2 IgG-scFv	17.8	148.8	0.13 ± 0.006

^a^TSP: Mean TSP values based on data from three independent experiments ± SEM.
