# Supplementary material for: Enhanced tumor-targeting selectivity by modulating bispecific antibody binding affinity and format valence
Source: Sci Rep. 2017 Jan 9;7:40098. doi: 10.1038/srep40098 (PMC5220356; doi:10.1038/srep40098)
Supplement: Supplementary Information [file srep40098-s1.pdf]

Supplementary Information

Enhanced tumor-targeting selectivity by modulating bispecific antibody binding affinity and format valence

Yariv Mazor[1,*], Kris F. Sachsenmeier[2#a], Chunning Yang[1], Anna Hansen[3], Jessica Filderman[2], Kathy Mulgrew[2], Herren Wu[1] and William F. Dall'Acqua[1,*]

[1]Departments of Antibody Discovery and Protein Engineering, MedImmune, Gaithersburg, MD, USA. [2]Departments of Oncology, MedImmune, Gaithersburg, MD, USA. [3]Departments of Respiratory, Inflammation and Autoimmunity, MedImmune, Gaithersburg, MD, USA.

#a Present address: Department of Translational Sciences, AstraZeneca, Waltham, MA, USA.

* Correspondence and requests for materials should be addressed to Y.M. (e-mail: mazory@medimmune.com), W.F.D.A. (e-mail: dallacquaw@medimmune.com)

a **Human HER2(exon 5)**

```
GCAGGGCTACGTGCTCATCGCTCACAACCAAGTGAGGCAGGTCCCACTGCAGAGGCTGCGGATTGTGCGAGGCACCCAGCTCT
TTGAGGACAACTATGCCCTGGCCGTGCTAGACAATGGAGACCCGCTGAACAATACCACCCCTGTCACAGGGGCCTCCCCAGGA
GGCCTGCGGGAGCTGCAGCTTCGAAGCCTCACAG
```

b **HER2.ko clone #3-2**

```
CCAGCTCTTTGAGGACAACTATGCCCTGGC-GTGCTAGACAATGGAGACCCGCTGAACAATACCACCCCT   (1 bp deletion)
CCAGCTCTTTGAGGACAACTATGCCCTGGC..CGTGCTAGACAATGGAGACCCGCTGAACAATACCACCCCT (128 bp insertion)

      TGCGGCGACCGAGTTGCTCTTGCCCGGCGTCAATACGGGATAATACCGCGCCACATAGCAGAAC
      TTTAAAAGTGCTCATCATTGGAAAACGTTCTTCGGGGCGAAAACTCTCAAGGATCTTACCGCTG
```

HER2.ko clone #54

```
CCAGCTCTTTGAGGACAACTATGCCCTGGC-GTGCTAGACAATGGAGACCCGCTGAACAATACCACCCCT   (1 bp deletion)
CCAGCTCTTTGAGGACAACTAT-------CCGTGCTAGACAATGGAGACCCGCTGAACAATACCACCCCT   (7 bp deletion)
```

HER2.ko clone #3-4

```
CCAGCTCTTTGAGGACAACTA-----------TGCTAGACAATGGAGACCCGCTGAACAATACCACCCCT   (11 bp deletion)
CCAGCTCTTT--------------------CGTGCTAGACAATGGAGACCCGCTGAACAATACCACCCCT   (20 bp deletion)
CCAGCTCTTTGAGGACAACTAT-------CCGTGCTAGACAATGGAGACCCGCTGAACAATACCACCCCT   (7 bp deletion)
```

Supplementary Figure S1: Genomic sequence analysis of NCI-H358.HER2.ko clones. **(a)** Genomic DNA sequence of human HER2 (exon 5). Green indicates gRNA and arrow the expected cleavage site. **(b)** Genomic sequence analysis of HER2.ko clones. Total DNA was extracted from single clones and PCR products flanking the cleavage site were sequenced to detect for genomic changes. Shown are the genotype sequences of each clone.

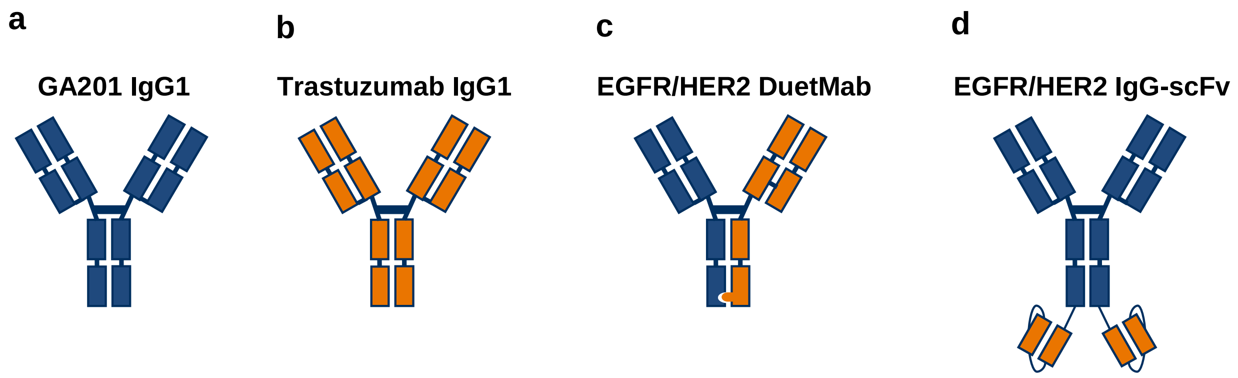

Supplementary Figure S2: Schematic diagram of antibody formats. **(a)** Bivalent monospecific anti-EGFR GA201 IgG1. **(b)** Bivalent monospecific anti-HER2 trastuzuman IgG1. (**c**) Monovalent bispecific EGFR/HER2 DuetMab. (**d**) Bivalent bispecific EGFR/HER2 IgG-scFv.

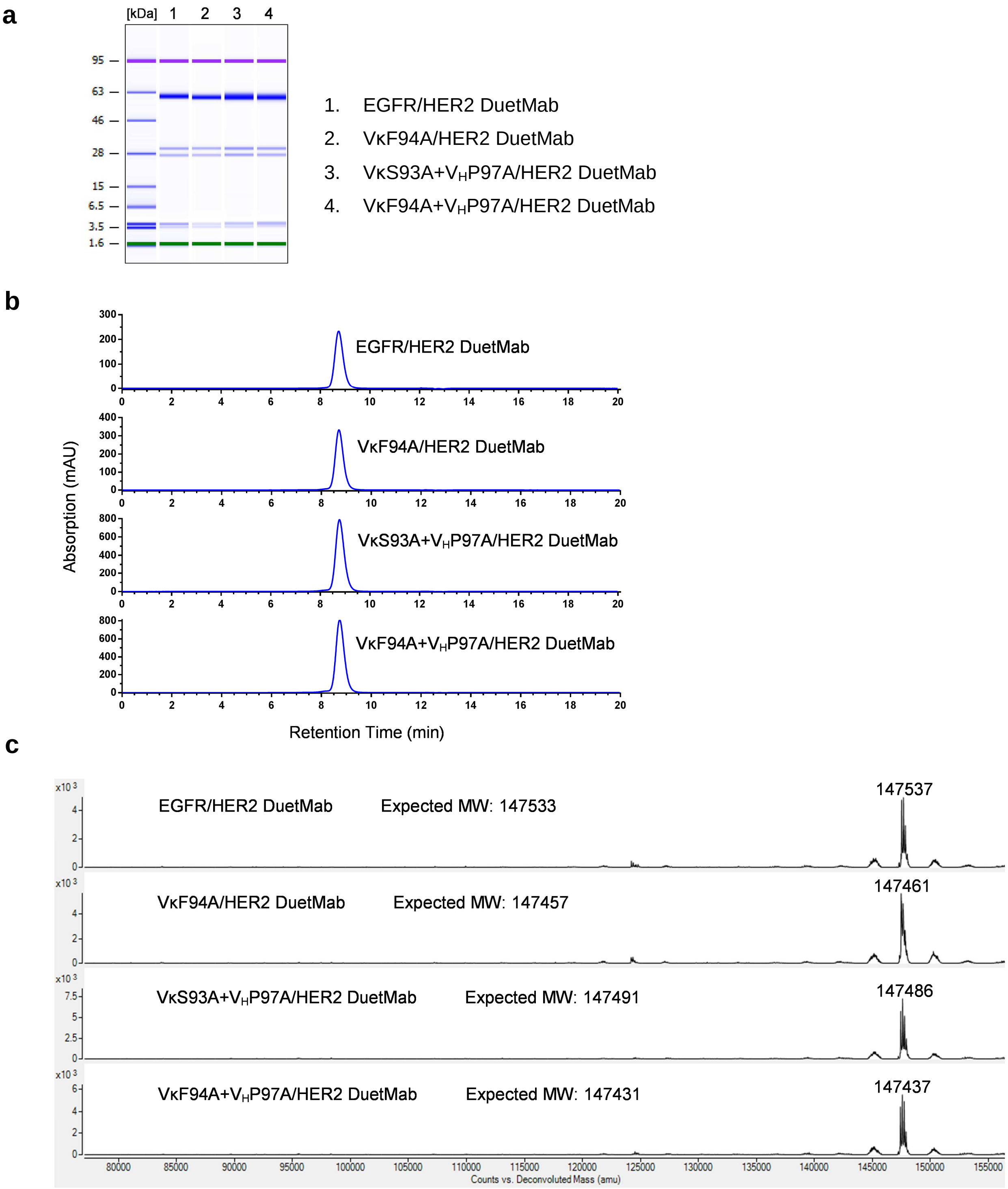

Supplementary Figure S3: Physico-chemical characterization of purified EGFR/HER2 DuetMab variants. (a) Capillary gel electrophoresis under non-reducing conditions using Agilent Protein 80 Chip. **(b)** Analytical size-exclusion chromatogram of intact EGFR/HER2 DuetMab variants. (**c**) Overlay of deconvoluted MS. Indicated is the theoretical and measured mass of intact EGFR/HER2 DuetMab variants.

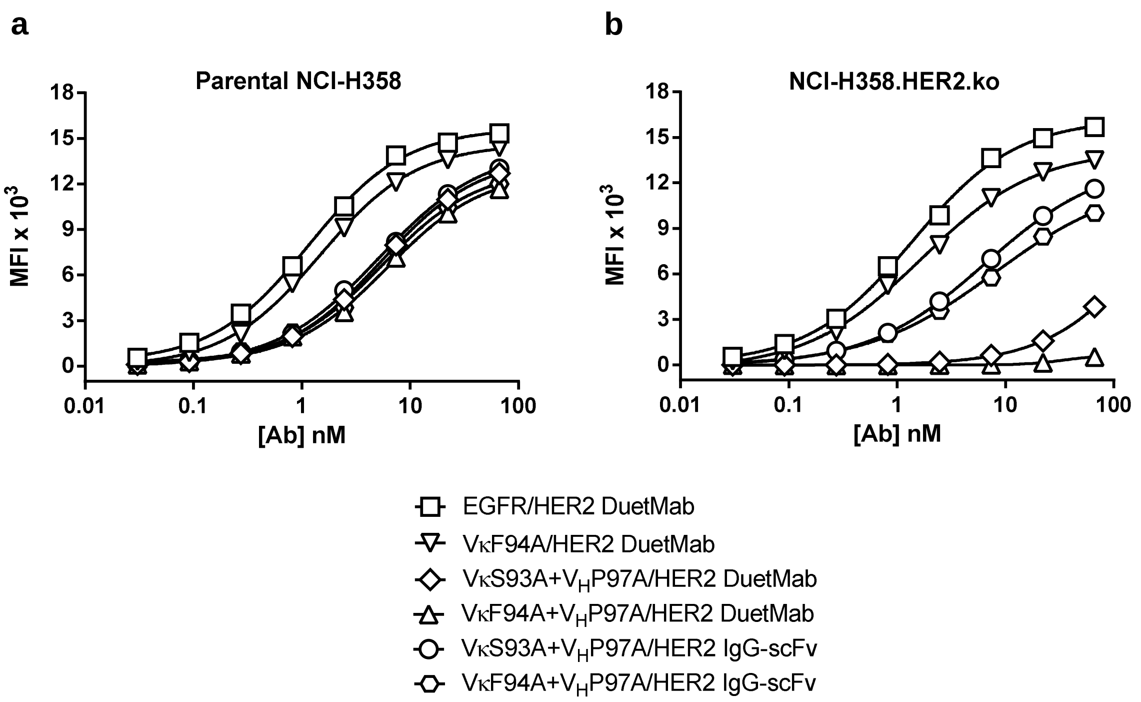

Supplementary Figure S4: Cell binding of EGFR affinity-modulated variants formatted as monovalent bispecific DuetMabs and bivalent bispecific IgG-scFvs. (a) Cell binding to parental NCI-H358 cells. **(b)** Cell binding to NCI-H358.HER2.ko cells. Half-maximal effective concentration (EC_{50}) values were used to estimate apparent cell binding affinities. Each point represents the mean values of triplicate wells and the ± standard error of the mean (SEM) is represented by error bars.
